# Lung clearance index in subjects with cystic fibrosis in Italy

**DOI:** 10.1186/s13052-019-0647-5

**Published:** 2019-05-02

**Authors:** Enrico Lombardi, Simone Gambazza, Ugo Pradal, Cesare Braggion

**Affiliations:** 1 Azienda Ospedaliero-Universitaria Meyer, Pediatric University Hospital, Viale Pieraccini 24, 50139 Florence, Italy; 2 Fondazione IRCCS Ca’ Granda Ospedale Maggiore Policlinico, Cystic Fibrosis Centre, Milan, Italy; 3 Department of Clinical Sciences and Community Health, Università degli Studi di Milano, Milan, Italy; 4 Fondazione IRCCS Ca’ Granda Ospedale Maggiore Policlinico, U.O.C. Direzione delle Professioni Sanitarie, Milan, Italy; 5 UO Pediatria Ospedale di Rovereto, APSS Trento, Trento, Italy

**Keywords:** LCI, Cystic fibrosis, MBW, Ventilation inhomogeneity, Preschool, Children, Lung function

## Abstract

The Lung Clearance Index (LCI) is an index derived from washout recordings, able to detect early peripheral airway damage in subjects with cystic fibrosis (CF) with a greater sensitivity than spirometry.

LCI is a marker of overall lung ventilation inhomogeneity; in fact, as pulmonary ventilation worsens, the number of tidal breaths and the expiratory volumes required to clear the lungs of a marker gas are increased, as documented by a greater value.

In the field of CF, LCI allows indirect investigation of the small airways (< 2 mm) the site where, from a pathophysiologic point of view, the disease begins due to the defect of the CF transmembrane-conductance regulator (CFTR) protein. Infant pulmonary function changes seem to occur before clinically overt symptoms of lower respiratory illness occur.

When performing the test, it is important to refer to the American Thoracic Society and European Respiratory Society consensus statements and apply a strict standardization.

In Italy the first tests were carried out in 2014 for research purpose and now approximately 10 centers are collecting data and are experiencing a consistency in repeating exams.

Currently in Italian centers children at pre-school age are the main target: in this population it is important to have a sensitive and feasible test, non-invasive, that can be performed at tidal volume without sedation, and requiring minimal cooperation and coordination, and that can be used longitudinally over time. Another target could be the transplanted subjects to detect early signs of lung function decline.

The content of this paper captures the experience and discussions among some of the Italian centers where LCI is currently used for research and/or in clinical practice about the method and the need to have a common approach.

The aim of this paper is not to describe the methodology of MBW, but to inform the pediatric community about the possible application of LCI in CF.

## Introduction

The multiple breath washout test (MBW) has been used for long time to measure static lung volumes during tidal breathing, with minimal cooperation and coordination needs. Recently the lung clearance index (LCI), an index derived from washout recordings, has proven to be able to detect early peripheral damages in subjects with cystic fibrosis (CF) with a greater sensitivity than conventional spirometry. This is a potential big opportunity for research and ultimately for clinical use.

The content of this paper captures the experience and discussions about the method and the need to have a common approach among some of the main Italian centers where LCI is currently used for research and/or in clinical practice.

The aim of this paper is not to describe the methodology of MBW, but to inform the pediatric community about the possible applications of LCI in CF, while many papers about the standardization are currently available in the literature.

### Lung clearance index: state of the art

The measurement of lung volume using inert gas dilution was reported first in the 1940s [1, 2]. The first respiratory mass spectrometer device was developed in 1952 [3] but the modern era of respiratory gas chromatography began in 1971 [4]. Initially MBW devices used 4% sulfur hexafluoride (SF_6_) as inert gas, but at present this concentration of the gas is generally no longer utilized, mostly because of concerns regarding greenhouse gas emissions and costs. Nitrogen wash-out devices measuring gas concentration via molar mass have been used since 1985 [5, 6]. For main stream molar mass inert gas concentration calculation, temperature and humidity corrections are necessary [7]. Different devices show similar performance in terms of discrimination power and reproducibility, but they cannot be considered as interchangeable [8]. Current data have not indicated any ethnic differences [9].

MBW devices measure how many lung volume turnovers (usually 6–7) are needed to wash out traces of an inert gas from the lungs. In the case of exogenous inert gases (SF_6_, He), the gas is released at a known concentration during the wash-in phase and the wash-in is complete when the expired gas concentration reaches the concentration of the gas supplied. On the contrary, no formal wash-in phase is required for nitrogen and few tidal breaths are carried out to ensure that the concentration of N_2_ is stable (normally 80%); during the washout phase, the subjects inhale gases that do not contain the tracer gas (room air for SF_6_ devices and 100% oxygen for N_2_ devices). The washout is interrupted once the tracer gas concentration reaches 1/40th of its starting concentration in 3 consecutive breaths [10, 11].

LCI is a marker of overall lung ventilation inhomogeneity; in fact, as pulmonary ventilation worsens, the number of tidal breaths and the expiratory volumes required to clear the lungs of the marker gas are increased, as documented by a greater LCI value [12].

Over the past 15 years, the application of LCI in CF has become widespread. In the field of CF, LCI allows indirect investigation of the small airways (< 2 mm); the site where, from a pathophysiologic point of view, the disease begins.

### Cystic fibrosis

Unlike the conducting airways, where mucosal biofilm is removed by ciliary movements, in the smaller airways the liquid must be reabsorbed, otherwise the exchange is hindered. The defect of CFTR (cystic fibrosis transmembrane-conductance regulator) protein explains the damage at the early stage of cystic fibrosis disease: the protein is expressed in the airway surface epithelium, that loses its absorption capacity, and in the subcutaneous glands, that secrete an altered mucus obstructing the glandular ducts [13, 14]. In addition, the pH of the membrane is affected by the impaired exchange of chloride and bicarbonate with serious consequences on innate immunity [13].

CF leads to obstructive clinical presentations, with ventilation inhomogeneity (VI), heterogeneous flow-volume loops and uneven topographic damage distribution [15].

Lung disease remains the main cause of morbidity and mortality in patients with CF therefore it would be crucial to assess early intervention strategies, before clinical symptoms become apparent, through suitable and sensitive tools.

Several instrumental approaches are available today to detect lung damage in the early stages of the disease.

Examination of broncho-alveolar lavage (BAL) in children under 2 years of age may detect positivity for *Staphylococcus aureus, Pseudomonas aeruginosa* as well as neutrophils and elastase positivity, thus testifying that infection and inflammation begin early [16]. Detected at 3 months of age, elastase positivity in BAL proved to be an air trapping and bronchiectasis risk indicator as confirmed by CT and MRI in the following 3 years [16].

Even forced (thoracic and abdominal) FEVs may detect early signs of the disease [17]. Further diagnostic tools include electrical impedance tomography [18] and Helium MRI [19], an index of inhomogeneous perfusion [20–22], which may provide further information as compared to MBW, where non-ventilated regions do not contribute to the inhomogeneity shown by MBW [23].

Infant pulmonary function changes seem to occur before clinically overt symptoms of lower respiratory illness occur [24]; in fact, structural changes in small airways start shortly after birth in CF, they accumulate over time and subsequently reach a certain functional threshold quantifiable using MBW. LCI has proved able to detect early lung injury in CF in a large longitudinal study [25]. Moreover, LCI showed to be indicative for the presence or absence of structural lung changes after 3 years in 87% of cases in a prospective, longitudinal study [26].

Spirometry is conventionally used in the assessment of CF lung disease, however FEV_1_ can remain within normal limits throughout childhood. LCI requires only passive co-operation and it is able to identify inhomogeneous ventilation in children with normal spirometry [27]; moreover, it is much more sensitive but less specific than FEV_1_ [28]. LCI discriminates infants with CF versus healthy subjects and it gains more sensitivity if it is considered together with FEV_1_ [29]. Abnormal LCI values in the preschool age range are stronger predictors than preschool FEV_1_ of subsequent abnormal school age FEV_1_ [30]. LCI can be considered as a test performed at rest that predicts some abnormalities appearing at exercise in CF children with normal spirometry as well [31].

Furthermore, baseline LCI allows predicting pulmonary exacerbations in young patients with CF with the increase of 1 z-score resulting in 12% increase in the number of exacerbations/year and correlates with the respiratory domain score at the CFQ-R (cystic fibrosis questionnaire-revised), a validated patient-reported outcome, even in the subgroup with normal FEV_1_ [32].

According to the experiences collected so far, LCI positioning is in mild pulmonary disease, for early changes detection and possibly for disease evolution monitoring, considering that the examination must be performed on lungs that are still functioning.

LCI within-subject variability is low and this strengthens the use of LCI to monitor lung disease progression in CF patients. An increase in LCI > 17% compared to previous LCI-measurement in clinically stable CF patients may therefore indicate early lung disease progression [33].

Furthermore, in the small group of patients with clinical and CT evidence of lung damage only in late adolescence or adult age, LCI may play a role as well: in particular when the result of FEV_1_ is within the lower limit of normal (LLN) [34] and/or there are no reductions of FEF_25_ (forced expiratory flow at 25% of FVC). It can be clearly stated that LCI cannot be used alone as an index of the severity of disease and doesn’t give any further information in grading the severity of obstruction. At this aim, conventional spirometry, in particular FEV_1_ should be considered as the reference.

Sensitive outcome measures to assess the efficacy of therapeutic interventions in patients with CF play a great role. LCI, but not spirometry was able to detect a treatment effect from hypertonic saline inhalation in patients with CF with mild disease [35]. Moreover, LCI is an indicator of improvement after intra venous antibiotic treatment and the higher the LCI value is at the beginning of the study the more it decreases after treatment [36].

LCI has proven to be a more effective and sensitive alternative than FEV_1_ to assess response to treatment with Ivacaftor in patients with CF who have the G551D-CFTR mutation and reduced lung function, but still normal spirometry [37]. Using z-scores to adjust for the known growth (i.e. height)-related decline in LCI during early childhood [38] increases the validity of results in evaluating treatment effects [24].

LCI has been also suggested as a surrogate for chest computed tomography to detect structural lung abnormalities in preschool and school age children with CF, however it cannot replace chest imaging using computed tomography to screen for bronchiectasis in this population [39].

A systematic review of the clinimetric properties of LCI demonstrates its reliability, validity and responsiveness coupled to an attractive feasibility profile [40]. Eventually LCI can be used for research purposes because smaller sample sizes are required compared to FEV_1_, which requires large samplings and long observation periods [41].

### Technical aspects in pediatric patients with CF

In recent years, the statements and guidelines of the international scientific societies about the use of tests like LCI have increased [10, 11, 42, 43].

Several MBW devices for LCI measure in children and adults [44] have been compared, but a deep analysis of different devices goes beyond the purpose of this paper. Exhalyzer® D is the device chosen by the European Cystic Fibrosis Society [8] and the one most frequently used in Italy. However, several software are available for analyses of MBW indices [45].

Hereinafter we are not going to describe in depth the technical aspects of MBW; for this purpose, it is important to refer to the American Thoracic Society and European Respiratory Society consensus statements when performing the test, for preschool age patients, a specific document has recently been published [43], for children and adults the 2013 guidelines should be followed [11].

**Fig. 1:**
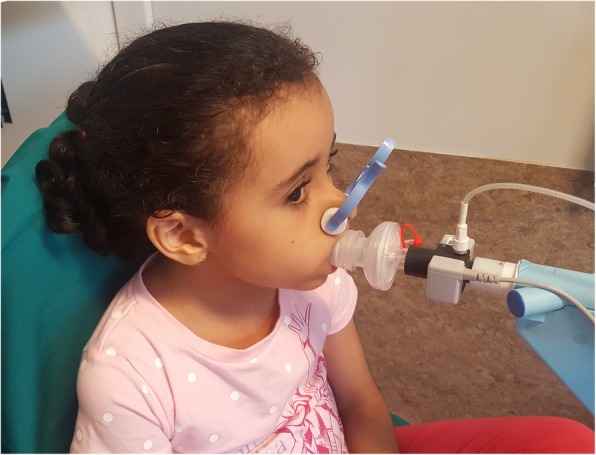
Measurement conditions. Preschool girl performing MBW while seating and breathing at tidal volume through the mouthpiece and a bacterial filter (permission to publication obtained)

The preschool subject should be tested in seated position breathing at a tidal volume through a mouthpiece or a mask closely adhering to the face (Fig.1). The tracer gas (helium, argon, or sulfur hexafluoride) is first washed in then washed out; alternatively, 100% oxygen can be inhaled to wash out the resident nitrogen from the lungs, without a wash-in phase.

There are many variables that need to be taken into account when performing the test:

- the stability of clinical condition
- the administration of bronchodilator therapy
- the possible execution of physiotherapy before examination, that can modify airway obstruction and ventilation inhomogeneity
- equipment calibration (depending on temperature, etc.)
- the stability of the wall mounted gas-system

In order to standardize all technical variables, it would be desirable to use the standard kit and all other materials provided by the manufacturer.

Criteria for technical acceptability are reported in the ERS/ATS Consensus [11] but there are several aspects that require adaptation to the preschool age [46]. The environment for preschool MBW testing should be as child-friendly as possible and distraction during the assessment must be enough to take the child’s attention away from his/her breathing. LCI success rates are 76–90% in infants during quiet natural sleep [47, 48] or with sedation [49].

In preschoolers, the use of a facemask, sealed with putty, and video distraction, achieves success rates of nearly 80%, though varying with age: 50% in 2–3 years old rising to 87% of 5–6 years old [50]. Similarly, LCI variability improves with age to a within-test CV (coefficient of variation) of 5.2% in preschoolers [50]. In a clinical study, it was possible to perform LCI examination in 90% of cases in the 4–16 age group, while 2 or more measurements were performed in 41% of cases [51].

According to the ERS/ATS consensus statement [43] at least three acceptable MBW trials whenever possible should be collected, however, in pre-school children data reported from only two acceptable trials provide comparable outcomes, ability to discriminate between health and disease, and associations with clinical markers of CF lung disease, improving feasibility in young children participating in longitudinal assessments and clinical trials [52].

LCI also requires some time to be performed, because repeated measurements may take several times to ensure reliable results. The reported average duration of a N_2_-MBW test in children with cystic fibrosis is 3.3 min (1.2 to 6.4), while it is 2.9 min (1.2–4.0) in controls [51].

The cross-contamination between patients can be minimized by cleaning and using disposable inserts [53] and following specific CF guidelines on infection prevention and control [54].LCI tests with different end-points (e.g. 1/30th, 1/20th or 1/10th of the initial concentration of tracer gas, or using a fixed washout period, for example 20s, or a fixed number of breaths) have been studied to save time in performing the test [55].

Reference values depend on the age of subjects, the type of device available, and the type of software used. Depending on the choice of gas, MBW indices differ substantially [44], for example, because helium is much lighter than Sulphur hexafluoride it generates higher LCI values [11].

Considering the age of subjects and factors such as gas, equipment, dead space, software, LCI is usually below 8.5 lung turnovers in healthy subjects [45]. Other relevant factors influencing MBW measures, are the posture during tests (supine or seated) [56], as well as sedation.

Reference values are not interchangeable [40] and few data for MBW measurements across different age groups are available [57].

LCI is thought to be independent of age, but recent evidences suggest that in the first 5 years of life LCI decreases in a nonlinear pattern as height increases; therefore, the use of fixed upper limit of normal (ULN) is not appropriate for children < 6 yr. [38].

The different algorithms used in the available software package to calculate LCI values could explain some false normal values in CF patients, highlighting the need for meticulous standardization not only of procedures but also of software algorithms [58].

### The Italian scenario

Eight centers are currently using LCI in Italy. The first tests were carried out in 2014 for research purpose and now some centers are collecting data and are experiencing a consistency in repeating exams, provided that they apply a strict standardization.

#### Indications

Pulmonary function tests play a key role in the management of CF in Italian centers, in children over 6 years of age. However, several matters hinder the same efficacy in the assessment of younger children and infants.

LCI looks as an attractive opportunity, because it is a sensitive, non-invasive and feasible test, particularly in the preschool age target when minimal cooperation and coordination are required during the test at tidal volume.

Currently, there is no sufficient evidence to recommend inclusion of LCI into the routine diagnostic evaluation and clinical monitoring of patients with CF, as stated by the Cystic Fibrosis Foundation in 2015 [42], but LCI may be considered as a tool to evaluate ongoing symptoms or monitor response to treatment, and as a measure in clinical research studies [41].

The primary use of LCI is to study CF patients of all ages without clinical or functional evidence of lung impairment to evaluate whether subtle lung disease is present. Currently the main target of LCI in Italian centers are children of preschool age. Children under 6 years of age have a developing respiratory system and a constantly evolving respiratory physiology, they experience frequent clinical episodes that may result in severe damage. Within this age group a particular subgroup is represented by children aged 2 to 6 years: they are too *old* to be sedated, but too *young* to actively cooperate [10]. In this age group spirometric parameters such as mid-expiratory flows (FEF_25_, FEF_50_ or FEF_75_) are not reliable. Therefore, it is important in the preschool group to have a sensitive test, to be performed without sedation, at tidal volume, and to be used longitudinally over time. Although it is a non-invasive test, LCI can be time-consuming and requires dedicated staff, therefore the recommended frequency should be every 6 months under clinical stable conditions.

Furthermore, in transplanted subjects it is crucial to detect early signs of lung function decline and LCI, performed at an increased frequency, can be used for early medical referral.

In these scopes of LCI, preschool children and transplanted subjects, a prospective data collection is needed to acquire a greater experience in measurement and maximize the advantages.

#### Interpretation of results

The LCI results are clearly influenced by biological variability and should be interpreted within the clinical history of the patient, taking into account many physiological variables and technical issues. Up to now, LCI calculated as 1/40th of starting end-tidal concentration of N_2_ (LCI 2.5%) is the most robust output of the N_2_-MBW test; the validity of its shortened version, i.e. LCI 5%, that is 1/20th of starting end-tidal concentration of N_2_, has not reached a consensus yet.

Table 1 shows three different N_2_-MBW reports, as available with Exhalyzer® D. The means and coefficient of variation (CV%) of three test results are displayed. Subjects A and B completed three reproducible runs, defined as a variation of FRC and LCI 2.5 values within 10%. Subject C performed a poor technically acceptable test, with an FRC CV% above 15% and therefore results were not considered valid.

**Table 1:**
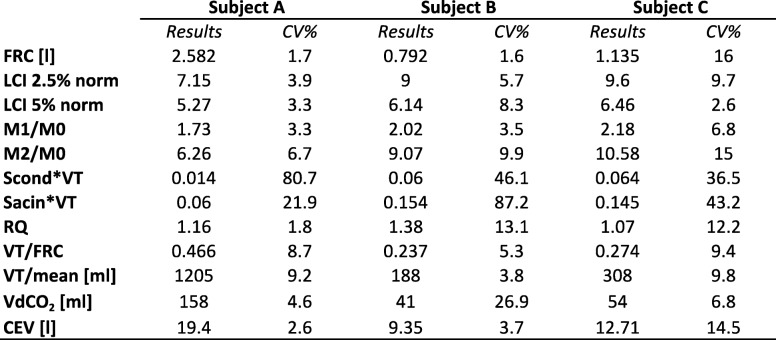
Standard N_2_-Multiple Breath Washout reporting available in Italian CF centers

CV = coefficient of variation; functional residual capacity; LCI 2.5% norm = normalized lung clearance Index at 2.5%; LCI 5% norm = normalized lung clearance index at 5%; M1/M0 = moment ratio: non-uniform alveolar flow volume distribution; M2/M0 = moment ratio: lung regions that empty late during the washout; Scond*VT = convection-dependent inhomogeneity corrected for tidal volume; Sacin*VT = diffusion-convection-dependent inhomogeneity corrected for tidal volume; RQ= respiratory quotient; VT = tidal volume; VdCO2 = volume of gas coming from dead space; CEV = cumulative expired volume

The first subject (A) is a 32 year-old man with CF, without pancreatic insufficiency and with normal pulmonary function (FEV_1_ equals to 95.1% predicted); LCI was 7.15 thus confirming an optimal respiratory functionality. Subject B is a boy with CF aged 5 years old, with an impaired pancreatic function. Classical spirometry was not available due to the young age, and therefore LCI was used as a mean of respiratory surveillance. Unfortunately, LCI was above the considered normality cut-off, describing an early lung disease otherwise not detectable. This information forced the CF team to further investigations. No conclusions could be drawn for subject C.

The understanding of other alternatives to LCI as outcomes of the N_2_-MBW test, such as the phase III analysis of the expirogram, i.e. Scond and Sacin, or moment ratios, i.e. M1/M0 or M2/M0, is beyond the scope of this report.

Interpretation and management of LCI results differ among centers. In order to manage the informative content of LCI tests, it might be useful to measure other parameters together with LCI, such as those coming from spirometry, when available, or from lung imaging, integrating the results from a clinical point of view, keeping in mind that different parameters are measuring different elements: some structural, some functional. MRI indices strongly correlated with LCI, as well as FEV_1_ [59]; on the other hand, LCI, but not body plethysmography, is associated with air trapping detected by CT scan [60].

LCI has been reported to be a useful marker to track early disease progression and based on the results of LCI more accurate decisions as the indication of other diagnostic tests, their early execution vs routine planned management and, last but not least, it may serve as a tool to guide therapies in young patients with CF [61, 62].

It should be highlighted that a good part of literature data has been obtained with SF_6_ and mass spectrometry, expensive equipment used in a few laboratories; therefore, there is the unmet need for new data to be acquired with the recently introduced instruments in children.

Health care professionals involved in LCI measurements are aware that a gold standard is unavailable, different methods have different reference values, different specificity and sensibility. In the Italian centers the limits of the exam are well-known, and so is the demand for working together in order to finalize a common behavior.

Recent studies have advanced our understanding of the role of the LCI in clinical practice: LCI is a promising surveillance tool to monitor early structural CF lung disease in preschool and school-age children although its exact clinical utility is still uncertain [63].

## Conclusions

LCI can bring added value to the evaluation of the patient’s clinical status. It is difficult to quantify this extra-informative content as it is extremely variable depending on the characteristics of each patient. It can be affirmed that the exam should be performed in the *right* patient to obtain meaningful data and to demonstrate its full value. Furthermore, LCI results should always be considered in the framework of symptoms, signs and other clinical exams to monitor disease severity over time and evaluate response to treatment.

In the preschool children group MBW test appears to provide the greatest discrimination, among the available lung functional tests, between children with CF and healthy control subjects, due to the regionally heterogeneous nature of early airway obstruction.

In Italy, time is probably ripe to create a specific LCI working group focusing on developing collaborative projects. A priority should be the implementation of a common data collection tool to minimize the differences among centers and obtain comparable tests. Device-specific reference values for preschool Italian children are also urgently needed.

## Abbreviations

ATS: American Thoracic Society
BAL: Bronchoalveolar Lavage
CF: Cystic Fibrosis
CFTR: Cystic fibrosis transmembrane-conductance regulator
CT: Computed tomography
ERS: European Respiratory Society
LCI: Lung Clearance Index
LLN: Lower Limit of Normal
MBW: Multiple Breath Wash out
MRI: Magnetic Resonance Imaging
MS: Mass spectrometry
SF_6_: Sulfur hexafluoride
